# EFhd2, a Protein Linked to Alzheimer's Disease and Other Neurological Disorders

**DOI:** 10.3389/fnins.2016.00150

**Published:** 2016-03-31

**Authors:** Irving E. Vega

**Affiliations:** Department of Translational Science and Molecular Medicine, College of Human Medicine, Michigan State UniversityGrand Rapids, MI, USA

**Keywords:** EFhd2, tau, neurodegenerative disease, neurological disorders, Alzheimer's disease

## Abstract

EFhd2 is a conserved calcium binding protein linked to different neurological disorders and types of cancer. Although, EFhd2 is more abundant in neurons, it is also found in other cell types. The physiological function of this novel protein is still unclear, but it has been shown *in vitro* to play a role in calcium signaling, apoptosis, actin cytoskeleton, and regulation of synapse formation. Recently, EFhd2 was shown to promote cell motility by modulating the activity of Rac1, Cdc42, and RhoA. Although, EFhd2's role in promoting cell invasion and metastasis is of great interest in cancer biology, this review focusses on the evidence that links EFhd2 to Alzheimer's disease (AD) and other neurological disorders. Altered expression of EFhd2 has been documented in AD, Parkinson's disease, Huntington's disease, Amyotrophic Lateral Sclerosis, and schizophrenia, indicating that *Efhd2* gene expression is regulated in response to neuropathological processes. However, the specific role that EFhd2 plays in the pathophysiology of neurological disorders is still poorly understood. Recent studies demonstrated that EFhd2 has structural characteristics similar to amyloid proteins found in neurological disorders. Moreover, EFhd2 co-aggregates and interacts with known neuropathological proteins, such as tau, C9orf72, and Lrrk2. These results suggest that EFhd2 may play an important role in the pathophysiology of neurodegenerative diseases. Therefore, the understanding of EFhd2's role in health and disease could lead to decipher molecular mechanisms that become activated in response to neuronal stress and degeneration.

## Introduction

*Efhd2* gene codes for a 26.8 kDa highly conserved protein, from nematodes to human, located in chromosome 4 (4E1;4 74.75 cM) in mice and chromosome 1 (1p36.21) in humans. EFhd2 was first identified in a proteomics screen designed to discriminate CD8 from CD4 and CD19 lymphocytes. The abundance of this novel protein was found to be reduced in CD4 and C19 in comparison to CD8 lymphocytes (Vuadens et al., 2004; Dütting et al., 2011). In this original study, the novel protein was named Swiprosin 1, in reference to the Swiss-Prot database used for the tandem mass spectrometry data analysis (Vuadens et al., 2004). Subsequently, the name of this novel protein was changed to EF-hand domain family, member D2 (EFhd2) due to the presence of two EF-hand calcium binding motifs. Characterization of EFhd2 demonstrated that it is an ubiquitous calcium binding protein, preferentially expressed in the central nervous system (Avramidou et al., 2007; Vega et al., 2008; Hagen et al., 2012; Ferrer-Acosta et al., 2013b). Further sequence analyses indicated that EFhd2 has a coiled-coil domain at the C-terminus, which is a conserved domain among fibrillar proteins and required for protein–protein interaction (Ferrer-Acosta et al., 2013a). At the N-terminus, EFhd2 has a distinctive polyalanine motif that varies in size (between 6 and 9 alanines) and it is only present in mammals (Dütting et al., 2011; Ferrer-Acosta et al., 2013a). The function of EFhd2's polyalanine motif is still unknown, but proteins containing polyalanine expansions have been shown to be linked to different neurological disorders (Albrecht and Mundlos, 2005). However, the physiological and pathological roles of EFhd2 are still poorly understood.

EFhd2 may function as a signaling or cytoskeleton regulatory protein. In WEHI231 cells, it was shown that EFhd2 is required for the regulation of the canonical NFkB pathway upon activation of the B-cell receptor (BCR; Avramidou et al., 2007; Hagen et al., 2012; Kim et al., 2013). ShRNA-mediated EFhd2 knockdown led to increase IkB phosphorylation, which is a prerequisite for translocation of NFkB to the nucleus, upon BCR activation (Avramidou et al., 2007; Kim et al., 2013). Based on this result, the authors suggested that EFhd2 may play a role as negative regulator of NFkB in the BCR signaling pathway (Avramidou et al., 2007). Alternatively, another study found EFhd2 at the plasma membrane, where it facilitates the assembly of the BCR and appears to work as a scaffold protein required for the function of Syk, SLP-65, and PLCγ2 during BCR-induced calcium flux (Kroczek et al., 2010). The functional interaction of EFhd2 with BCR signaling pathway and modulation of IkB phosphorylation indicates a potential regulatory role in cell survival or fate. Interestingly, EFhd2 was identified as a novel pro-caspase-9-interacting protein in H460 cells (Chęcińska et al., 2009). EFhd2 association with (inactive) pro-caspase-9 protein suggests that it may regulate the activation of apoptosis (Chęcińska et al., 2009). In contrast, other reports indicated that EFhd2 mediates actin bundling and regulates cell spreading and migration (Huh et al., 2013; Kwon et al., 2013). Moreover, recent studies indicated that EFhd2 was upregulated by epidermal growth factor signaling and mediates cell migration through the modulation of Rac1, Cdc42, and RhoA activity (Huh et al., 2015). Although, the direct role of EFhd2 in regulating cellular survival and apoptosis is still unclear, these results indicate that this novel protein plays an important role in modulating cellular responses elicited by different environmental cues, such as trophic factors or stimulating antibodies (Avramidou et al., 2007; Huh et al., 2015).

EFhd2 has also been associated with different pathological processes, from cancer to neurological disorders (Table 1). In cancer, EFhd2 was found to be overexpressed in the majority of carcinomas, colon cancer, and melanoma (Huh et al., 2015). The results indicated that EFhd2 may mediate invasion and metastasis of cancerous cells, suggesting it plays an important role in the biology of cancer. *EFhd2* gene expression and protein abundance have been also shown to be altered in AD and other neurological disorders. Thus, even though the role that EFhd2 plays in cancer biology is of great interest, this review focusses on EFhd2's role in the central nervous system, specifically its association with AD and other neurological disorders.

**Table 1:** **Identification of EFhd2 associated with neurological disorders**.

**Disorders**	**Disease**	**Results Summary**	**Method**	**References**
Movement	Parkinson's disease (PD)	EFhd2 was found to be secreted from microglia after exposure to nitrated α-syn	SELDI-TOF ProteinChip	Reynolds et al., 2008
		EFhd2 protein abundance was shown to be reduced in midbrain (*substantia nigra*) of PINK1-KO mice	2D-DIGE and Tandem Mass Spectrometry	Diedrich et al., 2011
		EFhd2 was identified as Lrrk2 interacting protein, suggesting a potential role in actin polymerization	Quantitative immunoprecipitation combined with knockdown (QUICK); Tandem Mass Spectrometry	Meixner et al., 2011
		EFhd2 increased expression correlates with IFN-γ and SNCA switch to positive co-expression in *substantia nigra* in PD cases gathered from four different datasets; relationship between inflammation and PD	Microarray	Liscovitch and French, 2014
	Amyotrophic Lateral Sclerosis (ALS)	EFhd2 was one of the proteins uniquely identified in lipid raft isolated from mouse overexpressing G93A mutant SOD1	2D-gel and Tandem Mass Spectrometry	Zhai et al., 2009
		EFhd2 was identified as a C9orf72 poly-GA co-aggregating protein	Immunoprecipitation and Tandem Mass Spectrometry	May et al., 2014
	Huntington's disease	EFhd2 down-regulation preceded phenotype onset and it was one of only nine identified proteins with sustained altered expression after onset in a mouse model for Huntington's disease	2D-gel and Tandem Mass Spectrometry	Zabel et al., 2009
Dementia and psychiatric disorders	Suicide	EFhd2 was found down-regulated in prefrontal cortex and up-regulated in amygdala of individuals that committed suicide	2D-DIGE and Tandem Mass Spectrometry	Kékesi et al., 2012
	Schizophrenia	EFhd2 protein was found up-regulated in dorsolateral prefrontal cortex samples from schizophrenia patients	Quantitative Mass Spectrometry	Martins-de-Souza et al., 2009
		EFhd2 protein was found up-regulated in postmortem mediodorsal thalamus samples from schizophrenia patients	Quantitative Mass Spectrometry	Martins-de-Souza et al., 2010
	Alzheimer's disease (AD) and related dementias	EFhd2 was shown to co-immunoprecipitate with tau proteins in temporal cortex derived from AD and FTDP cases and in brain samples from a tauopathy mouse model (JNPL3)	Immunoprecipitation and Tandem Mass Spectrometry	Vega et al., 2008
		EFhd2 is found overexpressed in AD (APP23) and stroke (pMCAO) mouse models	Microarray	Tseveleki et al., 2010
		Increased alternative splicing of EFhd2 in frontal cortex of AD patients in comparison to normal aging	RNA-Seq	Twine et al., 2011
		EFhd2 was found associated with tau aggregates in the somatodendritic compartment and co-purified with tau filaments; EFhd2 protein abundance also found increased in AD cases	Immunoblotting, immune-gold electron microscopy, and histology	Ferrer-Acosta et al., 2013b
		EFhd2 protein levels were found reduced in frontal cortices from different types of tauopathies and other dementias	Immunoblotting	Borger et al., 2014

## Is EFhd2 a modulator of functional synapse formation?

EFhd2 is highly expressed in neurons compared to other cell types of the central nervous system, where may play an important role in synapse formation (Reynolds et al., 2008; Vega et al., 2008; Ferrer-Acosta et al., 2013b; Borger et al., 2014). EFhd2 proteins were found in the cytosol and proximal to the membrane in neurons of most brain regions, including higher expression in the deeper layers of the cortex and hippocampus (Ferrer-Acosta et al., 2013b; Borger et al., 2014). EFhd2 co-localized with neurite markers such as tau, MAP2, synapsin, and PSD95, suggesting that its neuronal function could be associated with vesicle transport and synapse homeostasis (Borger et al., 2014; Purohit et al., 2014). Consistent with this putative function, a recent study showed *in vitro* that knockdown of EFhd2 increased synpasin 1a/b puncta labeling in neurites, suggesting that modulation of EFhd2 affects the development of functional synapses, but it had no effect on converting them to mature synapses as determined by the co-localization of synapsin and PSD95 (Borger et al., 2014). These results imply that EFhd2 may modulate the formation of new synapses, a process that it is relevant for different brain functions, such as learning and memory.

Characterization of an *Efhd2* knockout mouse provided further insights about its function in the central nervous system. Purohit et al. (2014) showed that deletion of *Efhd2* gene has no detectable effect on brain anatomy or function. Interestingly, they showed that vesicle transport velocity was enhanced in *Efhd2*^(−∕−)^ knockout primary hippocampal neurons and that EFhd2 protein inhibited kinesin mediated microtubule gliding *in vitro* (Purohit et al., 2014). The authors proposed that EFhd2 may interfere with the interaction between kinesin and microtubules (Purohit et al., 2014). In this regard, EFhd2 has been showed to co-purify with tubulin in the synaptosome fraction as well as to mediate actin bundling (Huh et al., 2013; Kwon et al., 2013; Purohit et al., 2014). Although, there is no evidence that *Efhd2* gene knockout affects brain function, these results suggest that EFhd2 may serve as a modulator of synapse formation by regulating the velocity of vesicle transport and cytoskeleton rearrangement.

## EFhd2 in Alzheimer's disease

EFhd2 protein abundance was found altered in AD, suggesting that *Efhd2* gene expression may be regulated in response to neurodegeneration (Vega et al., 2008; Borger et al., 2014; Table 1). Two different studies showed that the chromosome region encompassing the *Efhd2* gene locus is linked to (and a third study showed that it is associated with) late-onset Alzheimer's disease (LOAD; Hiltunen et al., 2001; Myers et al., 2002; Holmans et al., 2005). The linkage between *Efhd2* gene and LOAD is yet to be determined; however, we and others have shown that EFhd2 expression and protein abundance is altered in AD and animal models that mimic the pathophysiology associated with AD (Vega et al., 2008; Tseveleki et al., 2010; Borger et al., 2014). Previously, we showed that EFhd2 protein is increased in the tauopathy mouse model JNPL3, which expresses the human P301L mutant tau protein (Vega et al., 2008; Ferrer-Acosta et al., 2013b). We showed that EFhd2 protein abundance increased as the accumulation of pathological tau and progression of the motor impairment phenotype augmented in JNPL3 mice (Vega et al., 2008). In an independent expression profiling study, using a different AD mouse model, APP23, EFhd2 was also found to be overexpressed (Tseveleki et al., 2010). It is important to mention that the APP23 AD mouse model overexpresses human Amyloid Precursor Protein with double Swedish mutation (K670M/N671L) and it has been shown that tau mediates the toxicity observed in this mouse model (Sturchler-Pierrat et al., 1997; Ittner et al., 2010). These results suggest that EFhd2 is up-regulated in response to pathological processes associated with tau-mediated neurodegeneration.

EFhd2 protein increased abundance in mouse models of neurodegeneration was validated in postmortem AD brain (Vega et al., 2008; Ferrer-Acosta et al., 2013b). Using quantitative immunoblotting, we demonstrated that EFhd2 protein abundance is increased in postmortem frontal cortices in AD cases (Ferrer-Acosta et al., 2013b). In contrast, Borger et al. (2014) showed that EFhd2 protein abundance is reduced in AD cases and other dementias, including frontotemporal lobar degeneration with TDP43 pathology. Moreover, they showed that EFhd2 mRNA was also significantly lower in AD, when compare to normal aging controls. The discrepancy between these two reports could be due to differences in postmortem brain sample selection, agonal stage, activated glial cells, and/or differential immune cell infiltration. For example, in our study, we included only AD postmortem brain with Braak stage IV or higher (Ferrer-Acosta et al., 2013b). In Borger et al. (2014), Braak stage is reported for only a few of the cases used. It is important to mention that in those reported cases with Braak stage VI, EFhd2 protein level is higher than in the other AD cases that the Braak stage was not indicated. Other technical reasons, such as anti-EFhd2 antibodies used, protein extraction method and differential subcellular localization, could contribute to the discrepancy. Interestingly, Twine et al. (2011) identified EFhd2 as one of the genes with increased alternative splicing in frontal cortices of AD patients. This is consistent with previous results that identified two protein bands corresponding to EFhd2, which might contribute to the detection of different EFhd2 isoforms depending on the antibody used (Avramidou et al., 2007; Vega et al., 2008). Nevertheless, what is consistent among these studies is that EFhd2 expression was found altered in AD. Further studies are required to determine the molecular mechanisms involved in the regulation of the *Efhd2* gene and protein under pathological or cellular stress conditions.

**Figure 1:**
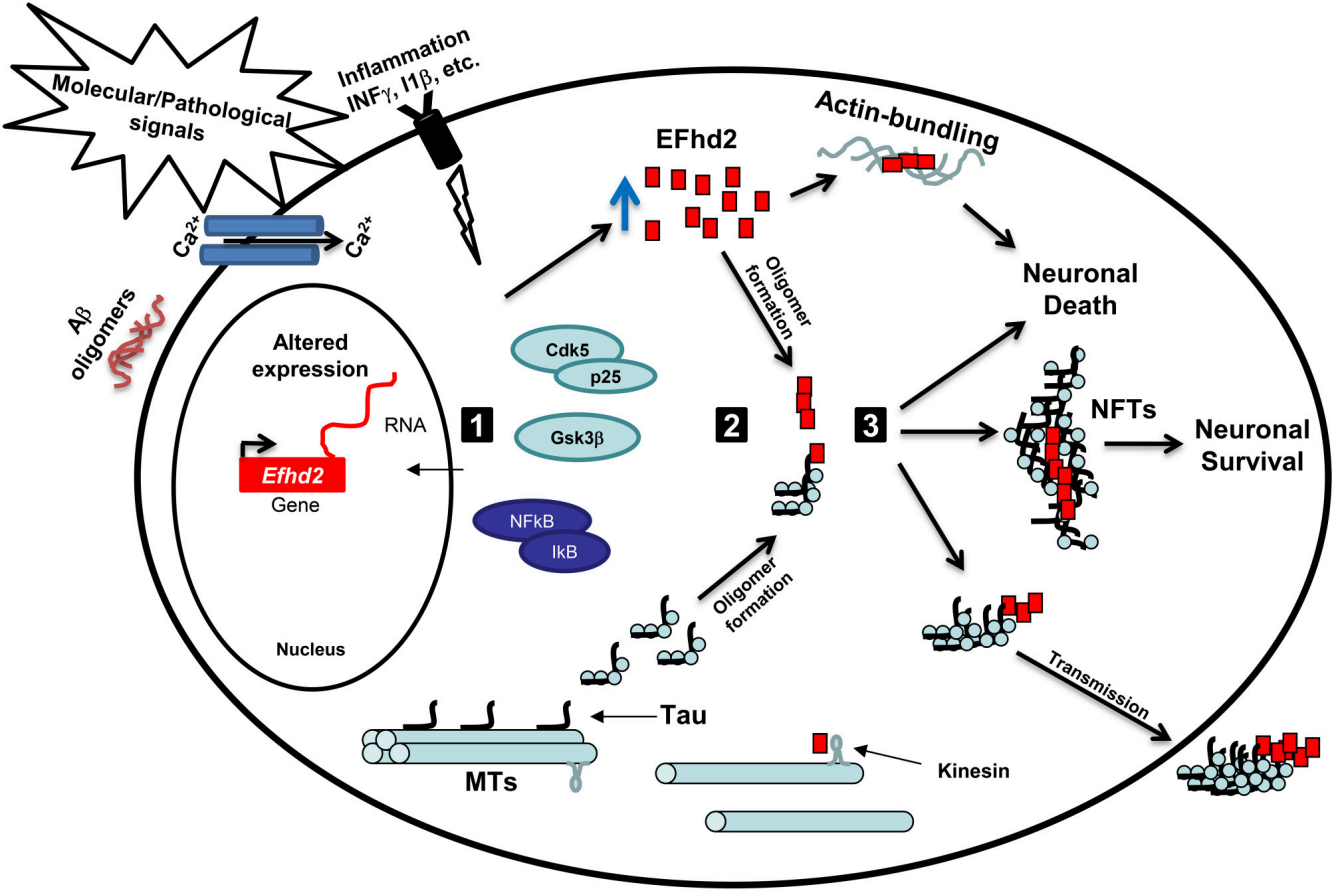
**Working hypothesis: EFhd2 role in neurodegeneration**. (1) Activation: Pathological signals, such accumulation of Aβ oligomers, calcium influx, and neuroinflammation, lead to the activation of specific kinases, such as GSK3β and Cdk5/p25, and upregulation of gene expression. These kinases mediate the hyperphosphorylation of tau proteins and promote its release from the microtubules (MTs). Altered expression of *Efhd2* gene leads to increase abundance of EFhd2 protein. (2) Oligomerization: The accumulation of EFhd2 proteins and its self-oligomerization properties could serve as a nucleation factor for hyperphosphorylated tau proteins, enhancing the kinetics of the formation of tau oligomers and/or neurofibrillary tangles at the somatodendritic compartment. Additionally, the accumulation of EFhd2 proteins affects kinesin-mediated fast axonal transport and promotes actin-bundling. (3) Toxicity: The accumulation of EFhd2 and tau oligomers leads to neuronal death and its release to the interstitial fluid, where are up-taken by neighboring neurons. Alternatively, EFhd2 could accelerate the transition from tau oligomers to neurofibrillary tangles (NFTs) as a neuroprotective mechanism, reducing the capacity of toxic tau transmission. Further studies are required to define the pathological role of EFhd2 in AD and other neurological disorders.

Further biochemical characterization of EFhd2 protein demonstrated that this novel protein has the molecular and structural features of amyloid proteins (Ferrer-Acosta et al., 2013b). *In vitro* studies showed that Thioflavin S (a dye that selectively binds to amyloid structures) binds recombinant EFhd2 protein, indicating that EFhd2 transition from a mostly helical and random coil structure to cross-beta-sheet (Ferrer-Acosta et al., 2013a,b). Amyloid proteins tend to form oligomers and filamentous structures. Electron microscopy analyses confirmed that EFhd2 forms filaments *in vitro* without the requirement of a nucleation factor (Congdon et al., 2008; Ferrer-Acosta et al., 2013b). Furthermore, the presence of calcium reduces EFhd2's ability to form filaments, and the coiled-coil domain was shown to be required for the formation of EFhd2 homodimers (Ferrer-Acosta et al., 2013b). These results indicated that formation of EFhd2 filaments could promote the association with pathological tau filaments. To test this hypothesis, immunohistological analyses of AD brain slices were performed. The results showed that EFhd2 co-localized with PHF1 (an antibody that recognized tau filaments) in the somatodendritic compartment, validating the association of EFhd2 with filamentous tau structures (Ferrer-Acosta et al., 2013b). Moreover, immune-gold electron microscopy showed that EFhd2 and tau, purified from AD brain, formed co-filaments (Ferrer-Acosta et al., 2013b). Moreover, *in vitro* protein–protein interaction assays demonstrated that EFhd2's coiled-coil domain is necessary for its association with tau proteins purified from brain extract derived from JNPL3 mice (Ferrer-Acosta et al., 2013b). Thus, it is plausible to hypothesize that formation of EFhd2 oligomers may serve as nucleation factor for tau oligomerization and, consequently, NFTs in AD and other tauopathies (Figure 1). Nevertheless, further studies are required to determine EFhd2's capability to enhance protein aggregation in tauopathy and other neurodegenerative disorders.

## EFhd2 is associated with Parkinson's disease and other neurological disorders

EFhd2 has been also found associated with Parkinson's disease (PD). Liscovitch and French (2014) showed that EFhd2 expression is increased in substantia nigra in PD. In this study, they found that EFhd2 overexpression correlated with the positive co-expression of α-synuclein and IFN-γ, establishing a molecular relationship between PD and inflammation (Liscovitch and French, 2014; Table 1). In contrast, EFhd2 protein abundance was found reduced in the substantia nigra of a mouse model where PTEN-induced kinase 1 (PINK1) has been knockout (Diedrich et al., 2011). PINK1 is a mitochondrial protein, the loss-of-function mutation of which induces early-onset PD. These conflicting reports could be due to the difference between mechanisms associated with sporadic PD cases and those underlying the phenotype of the PINK1 knockout mice. However, *in vitro* studies identified EFhd2 as a protein secreted/released by microglia cells upon incubation with nitrated α-synuclein (Reynolds et al., 2008). This is the first study that shows expression of EFhd2 in microglia cells, but, more importantly, it also indicates that EFhd2, a cytosolic protein, could be released from cells in response to a pathological signal. Taken together, EFhd2 altered protein abundance in PD provides strong evidence that it is involved in molecular mechanisms associated with neurodegeneration.

EFhd2 associates with known proteins linked to the pathophysiology of PD (Table 1). In a proteomics approach, EFhd2 was identified associated with leucine-rich repeat kinase 2 (Lrrk2), the most common causative gene of inherited PD (Meixner et al., 2011). Interestingly, this study demonstrated that Lrrk2 decreased actin polymerization, promoting the accumulation of monomeric actin (G-actin; Meixner et al., 2011). Knockdown of Lrrk2 affects actin cytoskeleton and cellular morphology, indicating that Lrrk2 plays a direct role on actin (Meixner et al., 2011). In contrast, EFhd2 promotes actin bundling (Huh et al., 2013). Thus, these results suggest that Lrrk2 and EFhd2 may compete for the interaction with F-actin, as regulators of actin cytoskeleton dynamics. Moreover, Lrrk2 has been shown to phosphorylate tau and promote tau aggregation, as observed in postmortem brain tissue from PD cases with Lrrk2 mutations (Guerreiro et al., 2015). On the basis of their link to neurodegeneration, it is reasonable to speculate that there could be a pathological connection between Lrrk2 and tau through EFhd2.

EFhd2 altered protein abundance and its association with pathological proteins have also been found in other neurological disorders. EFhd2 protein was identified as a co-aggregating protein with poly-GA C9orf72, a common pathogenic protein in amyotrophic lateral sclerosis (ALS) and frontotemporal lobar degeneration (FTLD; May et al., 2014). This result suggests that EFhd2 may be involved in the molecular mechanisms that lead to aggregation of pathological proteins, other than tau, in proteinopathies such as ALS. Consistently, EFhd2 was one of the uniquely identified proteins found in lipid rafts isolated from a mouse model that expresses the G93A SOD1 mutant; a mutation found in inherited ALS cases (Zhai et al., 2009). This result indicates that EFhd2 subcellular localization could also be altered in neurodegeneration.

In addition to its association with neurodegenerative diseases, *Efhd2* gene expression has also been found altered in psychiatric disorders. Two independent studies demonstrated that EFhd2 is up-regulated in schizophrenia (Martins-de-Souza et al., 2009, 2010). Postmortem analysis of dorsolateral prefrontal cortex and mediodorsal thalamus, two brain regions associated with the pathophysiology of schizophrenia, revealed a significant increase in EFhd2 protein level in comparison to normal control cases (Martins-de-Souza et al., 2009, 2010). In contrast, EFhd2 was found down-regulated in prefrontal cortex and up-regulated in amygdala of individuals that committed suicide (Kékesi et al., 2012). These results suggest that altered levels of EFhd2 are directly associated with brain pathology.

## Conclusion

In summary, EFhd2 harbors similarities to known and well-studied neuropathological proteins. For instance, EFhd2 is a structural disorder protein mainly composed of random coils and alpha helices, forms filamentous structures, associates with vesicle trafficking and cytoskeleton rearrangement, co-aggregate, and co-purify with pathological proteins, its expression and protein abundance is altered in neurodegenerative diseases. Based on these similarities and the published results discussed above, it is plausible to hypothesize that neuronal stress due to environmental cues (such as cytokines) or pathological protein aggregation induces changes in *Efhd2* gene expression (Figure 1). In this context, activation of signaling proteins, such as Cdk5 (Vázquez-Rosa et al., 2014), induces posttranslational modifications on EFhd2 that affect its calcium binding activity and promotes self-oligomerization (Figure 1). The accumulation of EFhd2 oligomers could serve as a nucleation factor of tau proteins facilitating its accumulation at the somatodendritic compartment, which also affects kinesin-mediated fast axonal transport (Figure 1; Kanaan et al., 2013). Consequently, the accumulation of EFhd2 proteins and tau aggregates would lead to activation of apoptosis (Figure 1). Interestingly, up-regulation of EFhd2 could also promote aberrant actin bundling, leading to the formation of Hirano bodies in AD (Figure 1, Sonoda et al., 2015).

Alternatively, EFhd2 altered expression in neurological disorders could be related to neuroprotection. The identification of EFhd2 co-localization with neurofibrillary tangles (NFTs) in the somatodendric compartment and its filament formation capability suggest that EFhd2 may affect the kinetic of tau filament formation, promoting the generation of stable ultrastructures (Figure 1). The generation of NFTs may sequester tau oligomers, preventing the spread of toxic tau to the interstitial fluid. Concomitantly, EFhd2's accumulation in NFTs induces a loss-of-function effect that promotes the generation of new functional synapses through increase vesicle transport velocity similar to that observed in *Efhd2* knockout mice. This may explain why NFT bearing neurons are still synaptically active and functionally integrated in neuronal circuits (Kuchibhotla et al., 2014). Nevertheless, further studies are crucial to determine the role that EFhd2 plays in the pathophysiology of neurological disorders.

## Author contributions

IEV revised the literature and wrote the manuscript.

## Funding

The work reviewed was supported, in part, by funding from National Institute of Neurological Disorders and Stroke (1SC1NS066988, 8R25NS080687).

### Conflict of interest statement

The author declares that the research was conducted in the absence of any commercial or financial relationships that could be construed as a potential conflict of interest.
